# Fabrication and Characterization of Monodisperse Magnetic Porous Nickel Microspheres as Novel Catalysts

**DOI:** 10.1186/s11671-015-1088-8

**Published:** 2015-10-05

**Authors:** Chao Teng, Jie He, Lili Zhu, Lianbing Ren, Jiwei Chen, Mei Hong, Yong Wang

**Affiliations:** Guangdong Provincial Key Laboratory of Nano-Micro Materials Research, School of Chemical Biology & Biotechnology, Peking University Shenzhen Graduate School, Shenzhen, 518055 China

**Keywords:** Magnetic microspheres, Porous nickel microspheres, Separable catalysts, 61.46.Hk, 75.75.-c, 81.07.-b

## Abstract

A facile and efficient hard-templating strategy is reported for the preparation of porous nickel microspheres with excellent uniformity and strong magnetism. The strategy involves impregnation of porous polymer microspheres with nickel precursors, calcination to remove the template, followed by thermal reduction. The morphology, structure, and the property of the Ni microspheres were characterized by scanning electron microscopy, X-ray powder diffraction, N_2_ adsorption-desorption isotherms, thermogravimetric analysis, and magnetic hysteresis measurement. The obtained porous nickel microspheres were monodispersed with a particle size of 0.91 μm and crystallite size of 52 nm. Their saturation magnetization was much higher than that of Ni nanoparticles. The unique porous nanostructured Ni microspheres possess catalytic activity and excellent recyclability, as demonstrated in the catalytic reduction of 4-nitrophenol to 4-aminophenol. The micropherical Ni catalysts could be easily separated either by an external magnetic field or by simple filtration.

**Electronic supplementary material:**

The online version of this article (doi:10.1186/s11671-015-1088-8) contains supplementary material, which is available to authorized users.

## Background

The development of nanoscience and nanotechnology has put forward a higher requirement to fabricate novel nanostructured catalysts with specific morphologies and functions [1–9]. A broad range of nanostructured metals such as Au, Ag, Fe, Cu, Pt, Pd, and Ni have shown enhanced catalytic properties for applications in organic synthesis [10–12]. Among different morphologies of metals, hierarchically porous spheres have attracted great research interests. Several reports demonstrated that assembling metal nanoparticles into porous hierarchical spheres led to improved properties over multiple-length scales [13–17]. Microspheres possess higher flowability and would not tend to agglomerate, which is inevitable for their nanoparticle counterparts. They also showed high catalytic efficiency derived from a high surface-to-volume ratio and maximized transport efficiency [18–22].

Porous nickel (Ni) catalysts have long been successfully employed in hydrogenation reactions, chemo-selective oxidative coupling of thiols, and Hantzsch condensation etc. [23–27]. Another important feature of Ni catalysts is their ferromagnetic properties. This offers possibility to easily remove the spent catalysts by magnetic field for the next reaction cycle.

The intrinsic properties of the Ni catalysts, such as catalytic activity and distinctive magnetic property, are deeply affected by their size, crystallinity, composition, and morphology [28–32]. Extensive efforts [33–40] have been devoted to fabricate various structures of porous Ni catalysts, making use of template synthesis, self-assembly, electro/chemical bath deposition, sol-gel method, and so on. The traditional RANEY® Ni, one of the most widely used Ni catalysts in industry, is prepared by chemical leaching and consists essentially of a porous skeletal structure exhibiting high catalytic activity. However, the fine powder nature of RANEY® Ni results in difficult catalyst separation from liquid phase reactions or plugging and pressure build-up in the fixed bed systems [41–45]. Recently, Fow’s group [46–48] prepared gauze-supported skeletal Ni catalysts which may be adapted to various reactor configurations by folding or stacking. However, supported catalysts are quite complex, and their fabrication approaches are time-consuming. For free standing Ni catalysts, Wu’s group [49] demonstrated a facile template- and surfactant-free method to prepare porous hierarchical Ni nanostructures by directly calcining the Ni-based flower-like precursor in Ar. Zhu et al*.* [50] developed a novel precursor hydrothermal redox method to fabricate the hierarchically porous structure of Ni hollow microspheres consisting of Ni nanoparticles on the shell. Yuan et al*.* [40] self-assembled synthesized porous Ni phosphate/phosphonate hybrid microspheres to combine the merits of organic and inorganic components. The hybrids showed catalytic activity for the reduction of 4-nitrophenol (4-NP) to 4-aminophenol (4-AP) due to the presence of Ni active sites on the pore surface. However, the Ni nanostructures produced from these soft-templating or template-free approaches were typically not uniform due to the undirected nucleation sites in the homogenous system.

In our previous works, we developed a hard-templating method utilizing monodisperse polymer microspheres to prepare uniform porous inorganic microspheres consisting of metal or metal oxide nanoparticles [51–54]. In this report, we applied the same principle to fabricate monodisperse porous Ni oxide microspheres by impregnation of porous polymer microspheres with Ni precursors followed by calcination to remove the template. Subsequent thermal reduction led to Ni microspheres. Different analysis techniques, such as scanning electron microscopy (SEM), X-ray powder diffraction, N_2_ adsorption-desorption isotherms, and magnetization curves were adopted to characterize the size, morphology, and magnetic property of the obtained microspheres. Their catalytic properties were also primarily studied.

## Methods

### Materials

The Ni precursor nickel acetate (Ni(Ac)_2_ · 4H_2_O) was purchased from Alfa Aesar. Ethylenediamine (EDA) was purchased from Sigma-Aldrich. The hard template porous polymer microsphere named poly(GMA-co-EGDMA) is a polymer of glycidyl methacrylate (GMA) cross-linked with ethylene glycol dimethacrylate (EGDMA) supplied by Nano-Micro Technology Company, China. 4-NP and NaBH_4_ used in the catalytic study of porous Ni microspheres were purchased from Sigma-Aldrich. Water was purified by distillation followed by deionization using ion exchange resins. Other chemicals were analytical grade and used without further purification.

### Preparation of Porous Nickel Oxide (NiO) and Ni Microspheres

Monodisperse porous NiO microspheres were fabricated by impregnation of porous poly(GMA-co-EGDMA) microspheres with Ni precursors followed by calcination to remove the template. In a typical synthesis, the poly(GMA-co-EGDMA) microspheres were firstly functionalized by EDA following our previous protocol [51]. Poly(GMA-co-EGDMA) microspheres of 10 g were dispersed in 240 ml water and sonicated for 0.5 h before 10 g of EDA was added, and the mixture was mechanically stirred at 80 °C for 13 h. The resulting EDA-functionalized poly(GMA-co-EGDMA) microspheres were washed repeatedly with distilled water till the filtrate was neutral, and the filter cake was dried at 50 °C. Afterwards, the EDA-functionalized poly(GMA-co-EGDMA) microspheres of 1 g were mixed with 0.5 g of Ni(Ac)_2_ · 4H_2_O, 6 ml of ethanol, and 4 ml of water. The mixture was then sonicated for 10 min and dried at 90 °C. Finally, the obtained composite microspheres were calcined at 600 °C for 12 h to form monodisperse porous NiO microspheres, which were subsequently reduced in a 95 % N_2_ / 5 % H_2_ atmosphere at 500 °C for 10 h to form porous Ni microspheres.

### Catalytic Study of Porous Ni Microspheres

The reduction of 4-NP by NaBH_4_ was chosen as a model reaction for investigating the catalytic performance of porous Ni microspheres. In a typical reaction, aqueous solution of 4-NP (5 mM, 1 ml) was mixed with fresh aqueous solution of NaBH_4_ (0.1 M, 5 ml) at room temperature. The microspheres (1.0 mg) were rapidly added into the reaction system. Subsequently, 1 ml of aqueous suspension was sampled at a given interval and filtered through a 0.45-μm membrane. The UV-visible absorption spectra of the filtrates were recorded at room temperature to monitor the reaction progress.

### Characterizations

Powder X-ray diffraction (XRD) of the synthesized microspheres was recorded using a Rigaku D/Max-2200PC diffractometer with Cu Kα at 40 KV, 200 mA. The crystallite size of the microspheres determined by XRD was calculated by the Williamson-Hall method. A field emission scanning electron microscope (SEM) Hitachi S4800 was used for the determination of the morphology and structure of the microspheres. The particle size of the microspheres was measured by a Beckman Coulter Counter size analyzer Multisizer 3. N_2_ adsorption-desorption isotherms were performed at 77 K on a Micromeritics Tristar 3020. The magnetic measurement of porous Ni microspheres was carried out at room temperature with a vibrating sample magnetometer under a varying magnetic field (ISOM, UPM, Madrid, Spain).

## Results and Discussion

### Preparation and Characterization of Porous Ni Microspheres

**Scheme 1:**
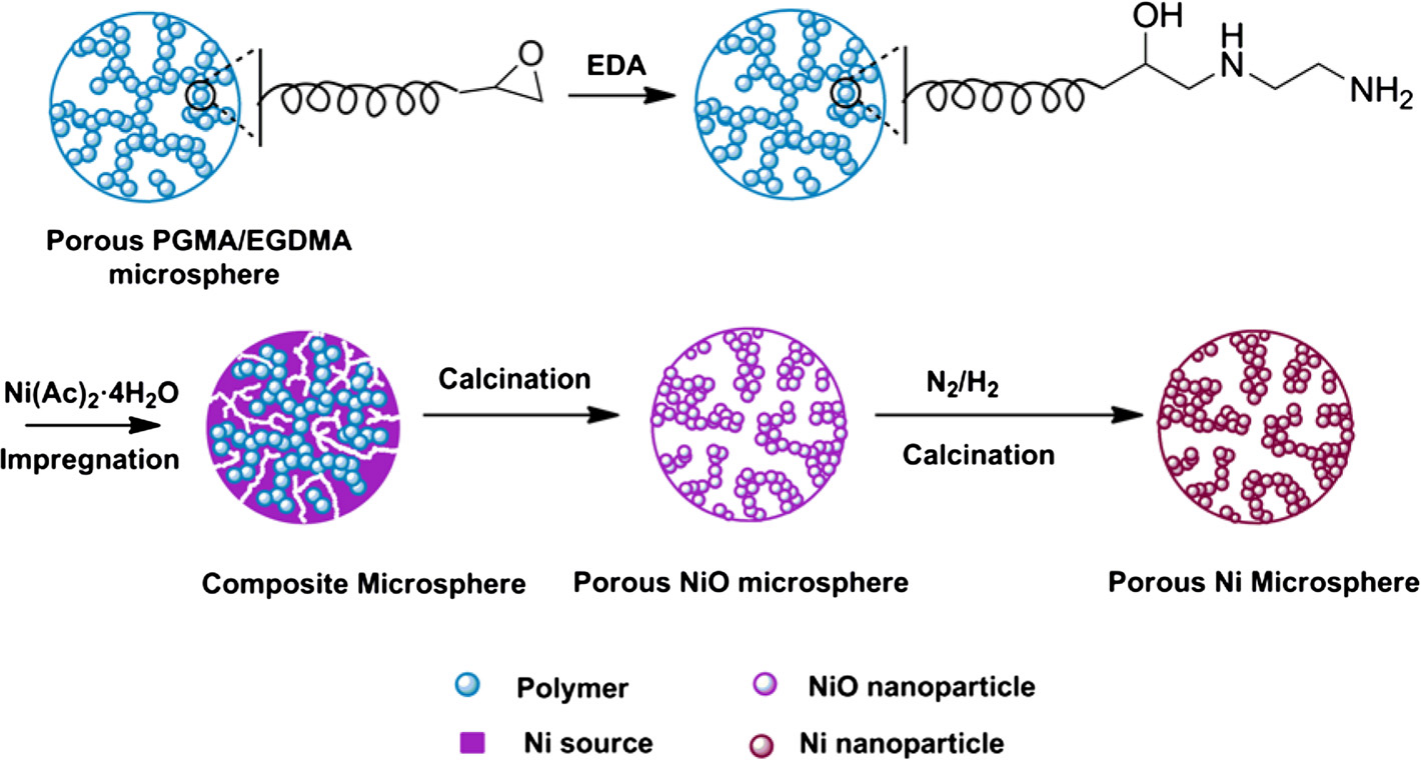
Schematic illustration of synthetic procedure for the porous Ni microspheres

In our previous work, monodisperse porous silica, magnetic nanoparticle-embedded silica microspheres, carbon microspheres, and zirconia microspheres have been successfully prepared using porous polymer microspheres as the hard template [51–55]. Notably, the unique pore structure and well-defined morphology of the parent polymer microspheres were inversely replicated into the obtained inorganic microspheres with similar monodispersity, which led to desired properties. This promising hard-templating method, as shown in Scheme 1, was used in this work to fabricate porous Ni microspheres. Porous poly(GMA-co-EGDMA) microspheres were chosen as the parent because of their easy functionalization due to the existence of surface epoxy groups. Functionalization by EDA introduced amino group to the porous poly(GMA-co-EGDMA) microspheres, which aided the impregnation of Ni precursor Ni(Ac)_2_ · 4H_2_O into the pore space. During the impregnation and drying process, the Ni precursors penetrated into the pores and incorporated into polymer microspheres to form composite microspheres. Calcination at 600 °C removed the polymer template, and at the same time oxidized the Ni precursor to NiO microspheres. Afterwards, the porous NiO microspheres were reduced in a hydrogen atmosphere to form porous Ni microspheres. Attempts to directly decompose the polymer in the Ni composite microspheres in a reducing hydrogen atmosphere to avert the step of forming NiO failed as the spherical structure collapsed due to the huge property differences between the polymer template and Ni.

**Fig. 1:**
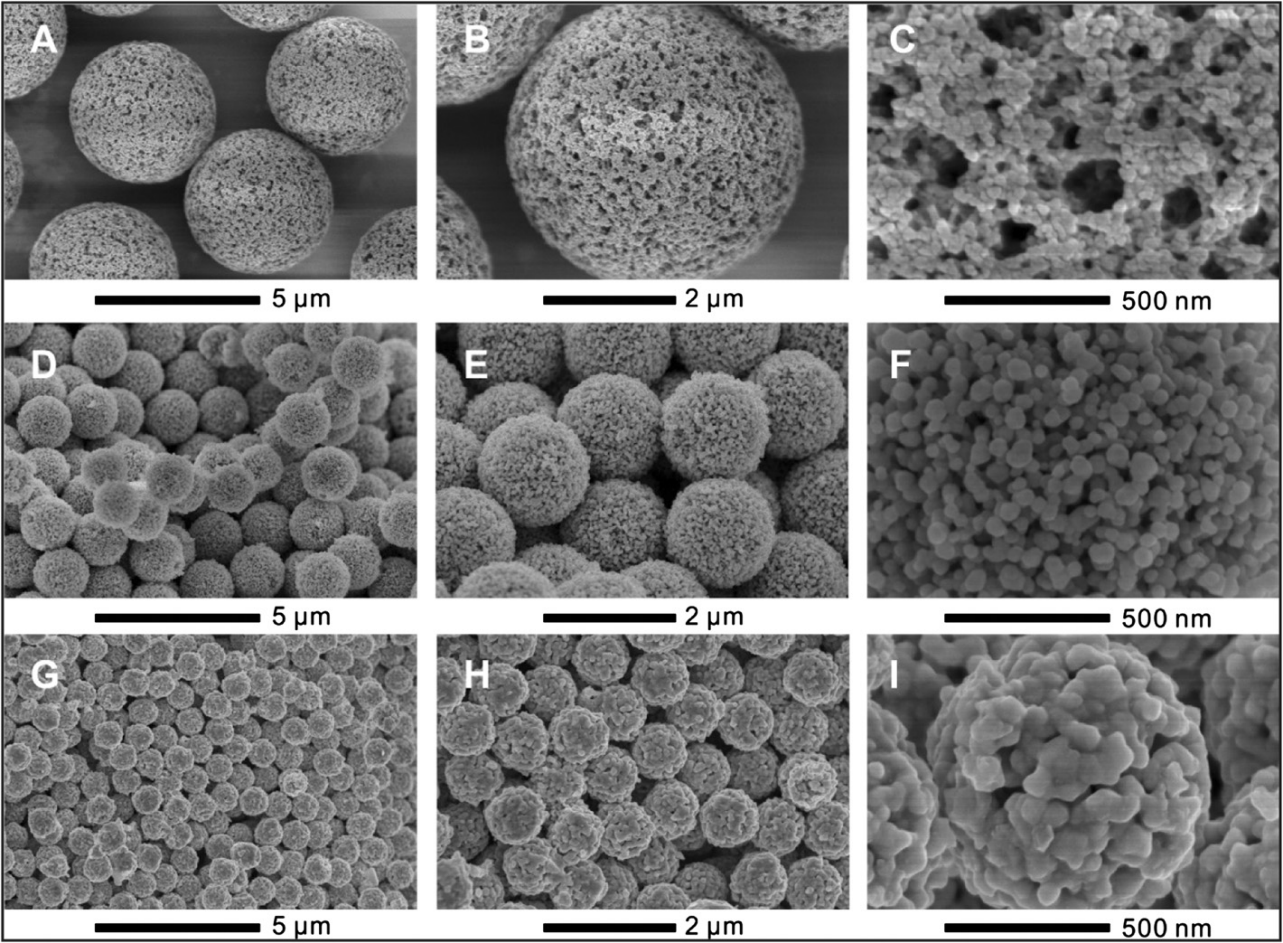
SEM images of (**a**–**c**) polymer microspheres, (**d**–**f**) NiO microspheres, and (**g**–**i**) Ni microspheres under different magnification

SEM images displayed in Fig. 1 demonstrate that the structured three-dimensional network of the parent polymer microspheres was well preserved in the synthesized porous NiO microspheres and porous Ni microspheres. Therefore, Ni precursors entered into the pores and interacted closely with the polymer skeleton. After reduction, the obtained porous Ni microspheres kept excellent monodispersity and well-defined spherical morphology. Due to the crystallite transformation at high temperature during reduction, the nanoparticles in Ni microspheres are larger than that of the NiO microspheres. As seen from the particle size of the polymer template and the synthesized microspheres in Table 1, the composite microspheres show similar size and monodispersity to the original polymer microspheres indicating that Ni precursor penetration did not cause structure deformation. Compared with the template microspheres (Fig. 1a–c), the porous NiO microspheres (Fig. 1d–f) were smaller probably due to shrinkage of the skeleton, high density of NiO, and growth of NiO crystallite size during calcination. High-temperature reduction further reduced the size of the formed Ni metal microspheres (Fig. 1g–i). All these as-prepared microspheres are in excellent independent spherical morphology. Agglomeration for the Ni microspheres, typically observed for molecular self-assembled ones (Zhu et al*.*, solid state sciences 2011;12:438–43), was not observed in our study. This demonstrated the strong templating power of our hard polymer microspheres, which counteract the surface chain-forming force caused by the magneto-static energy of ferromagnetic particles. The size distribution analyzed by the size analyzer confirmed the SEM observations (Table 1). The size of the original polymer microspheres and the polymer composite was very close, of 4.44 and 4.49 μm, respectively. Burning out the polymer template decreased the NiO microspheres to an average size of 1.83 μm, and further reductive process reduced the Ni microspheres to 0.91 μm. All microspheres were exceptionally uniform, and the coefficient of variation for particle size of NiO and Ni metal microspheres was 4.9 and 7.7 %, respectively.

**Table 1:** Properties of template and as-prepared microspheres^a^

Microspheres	Particle size (mean + SD) (μm)	Surface area (m^2^/g)	Pore size (nm)
Polymer	4.44 ± 0.11	75.33	21
Polymer/Ni	4.49 ± 0.11	58.26	21
NiO	1.83 ± 0.09	13.74	34
Ni	0.91 ± 0.07	2.55	42

^a^Particle sizes were determined by Coulter counter for polymer, polymer/Ni and NiO microspheres, and SEM for Ni microspheres; surface areas were determined using the Barrett-Emmett-Teller (BET) method, and average pore sizes were calculated using the Barrett-Joyner-Halenda (BJH) method

**Fig. 2:**
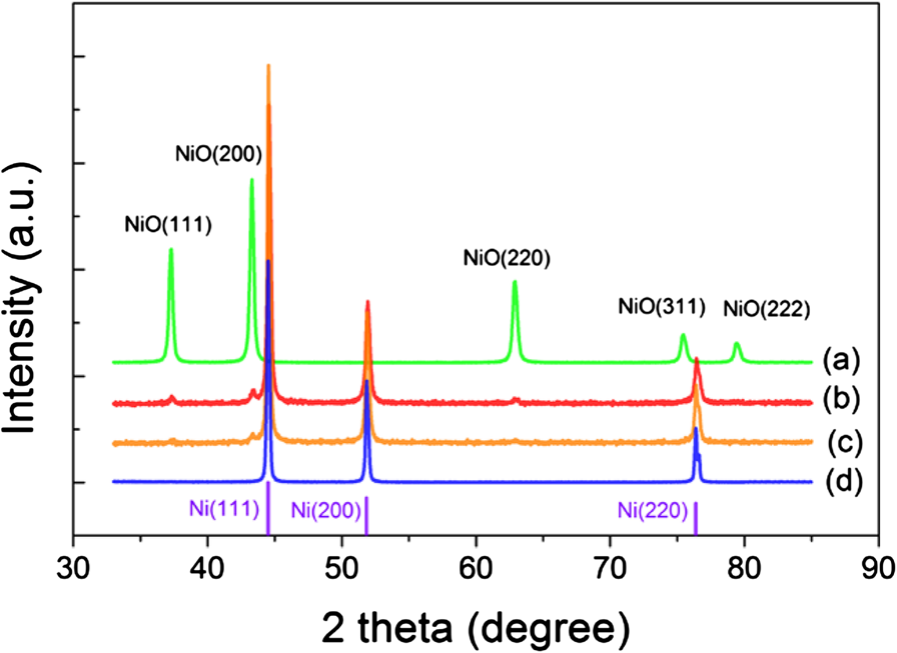
Wide-angle powder XRD patterns of porous NiO microspheres and Ni microspheres reduced at 300 °C, 400 °C, and 500 °C. The *violet lines* show the standard diffractions of Ni (JCPDS No. 04–0850)

The crystalline phases and the crystallite sizes of the powders were confirmed by XRD measurements. Powder X-ray diffraction patterns revealed that the obtained porous NiO microspheres and Ni microspheres are all crystalline. The reflection peaks of NiO microspheres (shown in Fig. 2), indexed to (111), (200), (220), (311), and (222), can be well-assigned to the cubic phase of NiO (JCPDS card no. 47–1049). The average crystallite size of porous NiO microspheres, calculated based on the Williamson-Hall method, was ca. 29 nm, consistent with the SEM observation (Fig. 1f). Reduction of porous NiO microspheres at 300 °C converted most of the microspheres into Ni as the diffraction peaks of the face-centered cubic (*fcc*) phase of crystalline Ni (JCPDS card no. 04–0850) with 2θ of 44.4°, 51.7°, 76.3°, corresponding to (111), (200), and (220) planes of crystalline Ni, becoming highly intense. However, the NiO peaks were still present. Increasing the reduction temperature to 400 °C reduced the impurity amount of NiO, and only with a reduction temperature of 500 °C, no NiO could be seen. The crystallite size of pure Ni microspheres was calculated to be ca. 52 nm according to the Williamson-Hall method, larger than that of the NiO microspheres. Obviously, high-temperature reduction gave rise to extensive sintering due to surface condensation. The sintering behavior could also be seen in the SEM images. Although reduction at 500 °C was essential for converting NiO to Ni thoroughly, this high thermal reduction temperature created a slightly higher degree of crystallite growth and more bicontinous interpenetration of the metallic particles. Fortunately, the Ni microspheres were still separated showing smooth surfaces and clearly discernable spherical interfaces.

**Fig. 3:**
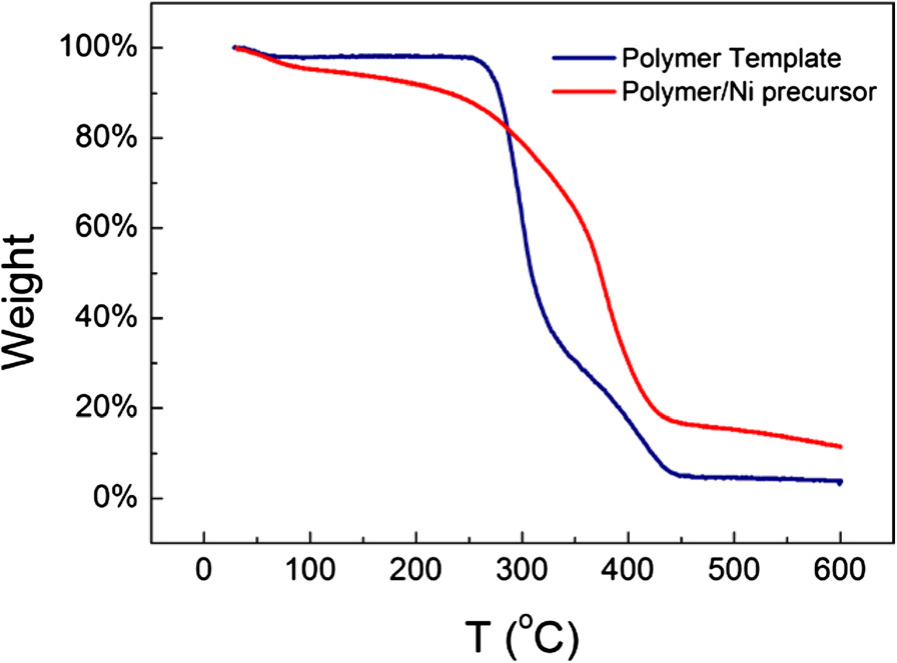
TGA curve of the polymer template and polymer/Ni precursor composite microspheres

The nitrogen adsorption-desorption results of the microspheres are presented in Table 1, and an additional file shows the curves of N_2_ adsorption-desorption isotherms and pore size distributions in more detail [see Additional file 1]. The template microspheres have a specific surface area of 75 m^2^ · g^−1^ and pore volume of 0.38 cm^3^ · g^−1^ with a BJH pore size of 21 nm. The composite microspheres exhibited a lower surface area of 58 m^2^ · g^−1^ indicating that Ni precursors successfully occupied the pore voids of the polymer microspheres. After calcination, the polymer part of the composite microspheres was removed. The complete removal of the polymer template was confirmed by thermogravimetric analysis (TGA) of the composite microspheres (Fig. 3). The polymer/Ni precursor composite microspheres underwent three stages of weight loss, 25–250, 250–450, and 450–600 °C. The weight loss of 11.8 % below 250 °C could be ascribed to the gasification of small molecules such as adsorbed water and ethanol. Between 250 and 450 °C, the decomposition of polymer chain, decomposition/dehydration of nickel acetate, and crystallite formation in the composite microspheres led to a weight loss of 71.4 %, obviously lower than the 92.7 % of template polymer microspheres, due to the remaining of NiO species. Little weight loss (2.8 wt %) was observed for calcination above 450 to 600 °C. Therefore, calcination at 600 °C could assure thorough burning away of the polymer skeleton.

**Fig. 4:**
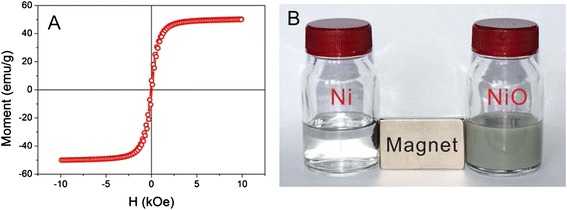
**a** Magnetic hysteresis curves of the porous Ni microspheres. **b** Photograph of microspheres before (NiO) and after (Ni) reduction under an external magnetic field within 10 s

Porous NiO microspheres containing both mesopores and macropores were obtained with a specific surface area of 14 m^2^ · g^−1^ and a pore volume of 0.1 cm^3^ · g^−1^. The lower surface area and pore volume of porous NiO microspheres compared to the template microspheres were probably due to the shrinkage of the skeleton and the concurrent growth of NiO crystallites during calcination, as well as the higher density of NiO. Thermal reduction process further reduced the as-synthesized porous Ni microspheres. The Ni microspheres obtained by 500 °C thermal reduction possessed a specific surface area of 2.6 m^2^ · g^−1^ and a pore volume of 0.01 cm^3^ · g^−1^ with BJH mesopores of 42 nm. In contrast to porous NiO microspheres, porous Ni microspheres showed lower surface area and pore volume but larger pore size owing to phase transformation upon high-temperature reduction. To confirm the magnetic property of porous Ni microspheres, its hysteresis curve was measured at room temperature and displayed in Fig. 4. The saturation magnetization (*Ms*), remnant magnetization (*Mr*), and coercivity (*H*_*c*_) were measured to be 50.26 emu · g^−1^, 4.58 emu · g^−1^, and 65 Oe, respectively, indicating the excellent magnetic property of porous Ni microspheres, as shown in Table 2 in comparison with other reported Ni nanostructures. The Ni microspheres prepared in this work exhibited much enhanced saturation magnetization than other reported Ni nanoparticles, suggesting better resistance to surface oxidation which are known to decrease the effective magnetic moment of Ni. The saturation magnetization of Ni microspheres is very close to that of bulk Ni, and the coercivity (H_c_) value is much lower, probably resulting from the shape anisotropy.

**Table 2:** Magnetism properties of Ni microspheres prepared in this study in comparison to other Ni structures

Sample	*Ms*/emu.g^−1^	*Mr*/emu.g^−1^	*Hc*/Oe	*Size*/nm
Ni microspheres	50.26	4.58	64.99	910 nm
Hollow Ni NPs [56]	21.1	0.69	32.3	300~450 nm
Ni nanoparticles [57]	32	5.0	40	12 nm
Bulk Ni [58]	55	2.7	100	–

### Catalytic Reduction of 4-NP by Porous Ni Microspheres

As one of the most common pollutants, 4-NP has attracted widespread attention. Many noble metal catalysts were devoted to catalyze the hydrogenation of 4-NP to obtain 4-AP which is a valuable intermediate for manufacturing anticorrosion drugs, antipyretic drugs, and analgesic. Herein, the catalytic performance of the fabricated monodisperse porous Ni microspheres for the hydrogenation of 4-NP was investigated. The reaction process was monitored by UV–vis absorbance at 400 nm.

**Fig. 5:**
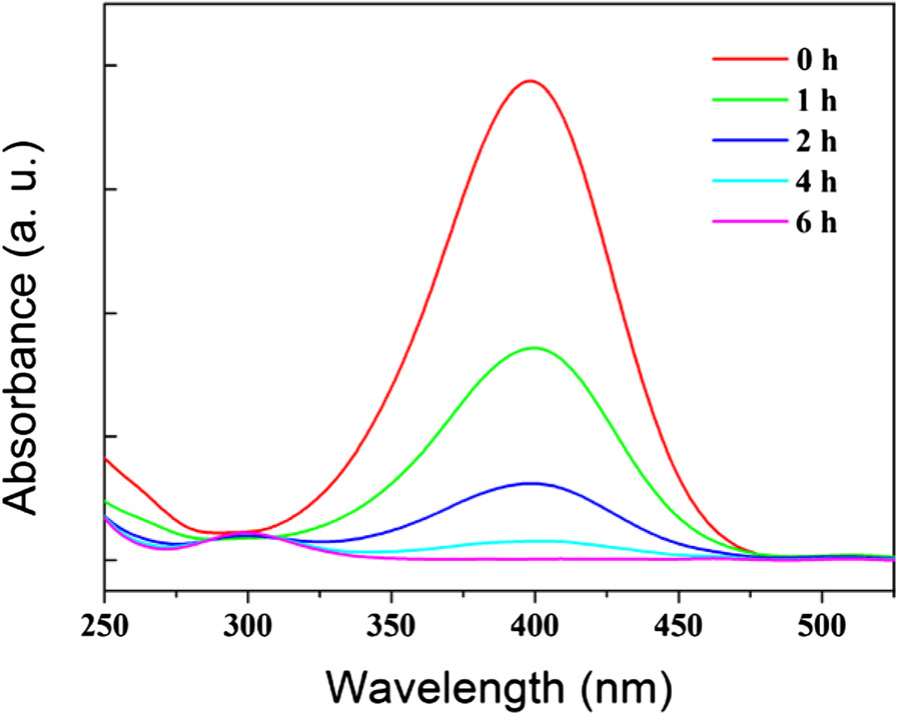
Time-dependent UV–vis spectrum of the reaction mixture for the 4-NP reduction reaction using the porous Ni microsphere as a catalyst

After addition of NaBH_4_ to the 4-NP solution, UV–vis absorbance changed from 317 to 400 nm due to the formation of 4-NPate. If no porous Ni catalyst was added, the absorbance at 400 nm remain unchanged revealing no reduction of 4-NP. When our porous Ni microspheres were added, the absorbance at 400 nm gradually decreased until no absorbance after 6 h, indicating complete reduction of 4-NP (Fig. 5). The initial bright yellow solution became colorless during the reaction course. The Ni microspheres can be easily recovered with a sand-core filter. When applying an external magnetic field, the Ni microspheres promptly transported to the wall of the reaction flask (Fig. 4), and the solution became transparent and easily separable.

## Conclusions

Uniform porous NiO and Ni microspheres were fabricated by employing porous polymer microspheres as the hard template. Both NiO and Ni microspheres exhibited well-defined morphology, excellent monodispersity, mesoporosity, and high crystallinity. Calcination to remove the polymer template for NiO microspheres and thermal reduction to produce Ni microspheres grew their crystallite sizes to 29 and 52 nm, respectively. The spherical particles were instilled in the micro-size range and no inter-particle agglomeration was observed. The Ni microspheres displayed outstanding magnetism with coercivity of 64.99 Oe, saturation magnetization of 50.26 emu · g^−1^, and remnant magnetization 4.58 emu · g^−1^, which are superior to other Ni nanoparticles. The Ni microspheres were catalytically active in the reduction of 4-NP, and the unique morphology and strong magnetism ensured convenient separation from the reaction mixture either by simple filtration or with an external magnetic field. The general strategy presented here holds potential to be applied to the design and fabrication of more metal or metal oxide materials with better physical or chemical properties for various applications.

## Additional file

Additional file 1: Figure S1. Data curves of N_2_ adsorption-desorption isotherms and pore size distributions. N_2_ adsorption-desorption isotherms (A) and pore size distributions (B) for polymer template, Polymer/EDA, Ni precursor, NiO and Ni microspheres. (PDF 168 kb)

## Abbreviations

GMA: glycidyl methacrylate
EGDMA: ethylene glycol dimethacrylate
EDA: ethylene diamine
4-NP: 4-nitrophenol
4-AP: 4-aminophenol
XRD: X-ray diffraction
SEM: scanning electron microscope
BET: Brunauer-Emmett-Teller
BJH: Barrett-Joyner-Halenda
