# Supplementary material for: Fabrication and Characterization of Monodisperse Magnetic Porous Nickel Microspheres as Novel Catalysts
Source: Nanoscale Res Lett. 2015 Oct 5;10:384. doi: 10.1186/s11671-015-1088-8 (PMC4593984; doi:10.1186/s11671-015-1088-8)
Supplement: Additional file 1: Figure S1. — Data curves of N2 adsorption-desorption isotherms and pore size distributions. N2 adsorption-desorption isotherms (A) and pore size distributions (B) for polymer template, Polymer/EDA, Ni precursor, NiO and Ni microspheres. (PDF 168 kb) [file 11671_2015_1088_MOESM1_ESM.pdf]

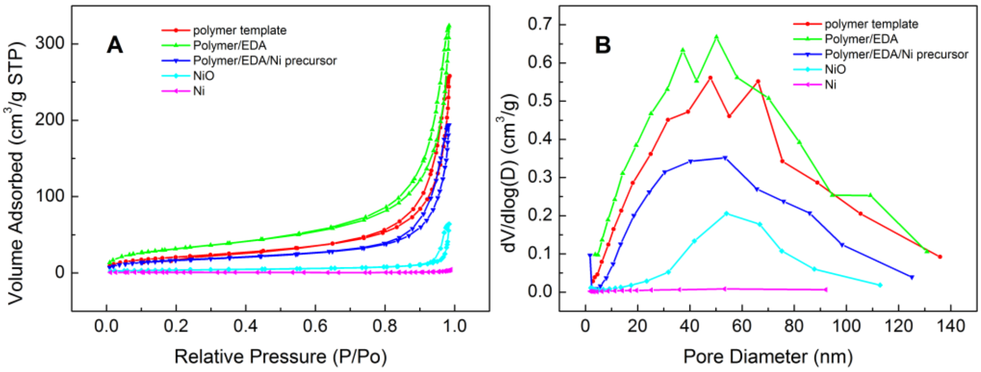

Figure S1. N_2 adsorption-desorption isotherms (A) and pore size distributions (B) for polymer template, Polymer/EDA, Ni precursor, NiO and Ni microspheres.
